# Impaired Top-Down Modulation of Saccadic Latencies in Patients with Schizophrenia but Not in First-Degree Relatives

**DOI:** 10.3389/fnbeh.2015.00044

**Published:** 2015-02-24

**Authors:** Simon Schwab, Miriam Jost, Andreas Altorfer

**Affiliations:** ^1^Department of Psychiatric Neurophysiology, University Hospital of Psychiatry, University of Bern, Bern, Switzerland

**Keywords:** schizophrenia, relatives, eye movements, saccades, saccadic latencies, state marker

## Abstract

Impaired eye movements have a long history in schizophrenia research and meet the criteria of a reliable biomarker. However, the effects of cognitive load and task difficulty on saccadic latencies (SL) are less understood. Recent studies showed that SL are strongly task dependent: SL are decreased in tasks with higher cognitive demand, and increased in tasks with lower cognitive demand. The present study investigates SL modulation in patients with schizophrenia and their first-degree relatives. A group of 13 patients suffering from ICD-10 schizophrenia, 10 first-degree relatives, and 24 control subjects performed two different types of visual tasks: a color task and a Landolt ring orientation task. We used video-based oculography to measure SL. We found that patients exhibited a similar unspecific SL pattern in the two different tasks, whereas controls and relatives exhibited 20–26% shorter average latencies in the orientation task (higher cognitive demand) compared to the color task (lower cognitive demand). Also, classification performance using support vector machines suggests that relatives should be assigned to the healthy controls and not to the patient group. Therefore, visual processing of different content does not modulate SL in patients with schizophrenia, but modulates SL in the relatives and healthy controls. The results reflect a specific oculomotor attentional dysfunction in patients with schizophrenia that is a potential state marker, possibly caused by impaired top-down disinhibition of the superior colliculus by frontal/prefrontal areas such as the frontal eye fields.

## 1 Introduction

Impaired eye movements are a well replicated finding in patients with schizophrenia, for a review see Levy et al. (1994) and Trillenberg et al. (2004). For example, scanpaths during exploratory eye movements are spatially limited and contain fewer fixations in patients with schizophrenia (Loughland et al., 2002; Minassian et al., 2005; Bestelmeyer et al., 2006; Benson et al., 2007; Nishiura et al., 2007; Takahashi et al., 2008; Suzuki et al., 2009; Elahipanah et al., 2011; Delerue and Boucart, 2013; Sprenger et al., 2013). When following a moving target, smooth-pursuit eye movements are impaired (Schwartz et al., 1995; Nkam et al., 2001, 2010; Nagel et al., 2012; Krishna et al., 2014). Further, more errors in antisaccade tasks were found (Fukushima et al., 1988; Petrovsky et al., 2009; Dyckman et al., 2011; Cutsuridis et al., 2014). Exploratory eye movements have been used as discriminator from controls, from other neurotic disorders, or from mood disorders in large-sample studies (Kojima et al., 2001; Suzuki et al., 2009; Benson et al., 2012). Impaired eye movements were also found in high-risk groups (Nieman et al., 2007; van Tricht et al., 2010), and fist-degree relatives (Holzman et al., 1974; Thaker et al., 2000; Radant et al., 2010; Kang et al., 2011; Aichert et al., 2013; Roberts et al., 2013), and people with schizotypal personality (O’Driscoll et al., 1998; Ettinger et al., 2005; Mitropoulou et al., 2011). Thus, impaired eye movements have been suggested to be an endophenotype (Allen et al., 2009) and to be related to genetics (Ross et al., 2002; Radant et al., 2010; Smyrnis et al., 2011), but not all of the various oculomotor parameters fully meet the criteria for a robust endophenotype (Calkins et al., 2008; Kallimani et al., 2009; Mazhari et al., 2011); this is not surprising due to the fact that they are associated with different cognitive processes and neural circuits (e.g., smooth pursuit, fixations, saccades, anti-saccades).

Already at the beginning of an eye movement, top-down processes can influence viewing behavior. Saccadic latency (SL) is the delay time after stimulus onset until a saccade is performed, due to the time required to program the motor command for a consecutive saccade with a specific direction and amplitude; this duration is usually around 200 ms. Studies of SL have shown that a task with higher cognitive demand (i.e., identification vs. simple observation) can reduce SL (Trottier and Pratt, 2005; Guyader et al., 2010). Shorter SL may be due to top-down disinhibition of the superior colliculus (SC), potentially mediated by the direct pathway connecting frontal/prefrontal cortex to the SC. However, previous studies that investigated the effect of tasks with various complexity on SL in patients with schizophrenia are sparse. In our previous study, we investigated top-down SL modulation by different visual tasks in schizophrenia and found impaired SL pattern compared to healthy controls (Schwab et al., 2013). However, familial association is an important criterion in the evaluation of endophenotypes and requires to expand studies with a group of first-degree relatives (Chen et al., 2006). Therefore, the purpose of this paper is to investigate the modulation of SL by higher- and lower-demand cognitive tasks in first-degree relatives and compare these data with SL pattern in healthy controls and patients with schizophrenia to evaluate SL as potential trait or state marker. We used two different visual tasks: color recognition (low demand) and orientation recognition (higher demand) to study SL patterns.

## 2 Materials and Methods

### 2.1 Participants

Ten first-degree relatives of patients with schizophrenia and 12 healthy controls underwent video-oculography during a visual task. Data of 13 patients and 12 healthy controls were used from a previous study (Schwab et al., 2013) and combined with the newly acquired data, resulting in a total dataset of 13 patients, 10 first-degree relatives, and 24 healthy controls. For demographic and clinical details, see Table 1. Patients either suffered from schizophrenia (10 paranoid, F20.0; 1 hebephrenic, F20.1) or acute polymorphic psychotic disorder (1 F23.0; 1 F23.1), according to ICD-10. All patients were recruited from the University Hospital of Psychiatry in Bern, Switzerland. First-degree relatives were unaffected parents of patients with schizophrenia who were recruited from a support group (Vereinigung der Angehörigen von Schizophreniekranken, VASK Bern). Healthy controls and first-degree relatives were not on any medications and had no history of schizophrenia or psychotic disorders. None of the subjects had a history of eye diseases, dichromacy, neurological diseases, diseases of the cervical spine, or shoulder/neck pain. From the newly acquired sample (controls and relatives), six subjects were excluded from analysis (four due to bad data quality, one due to cervical spine surgery, and one due to psychoactive medication). The study was approved by the ethics committee (Kantonale Ethikkommission Bern, No. 135/09). Written informed consent was obtained from all participants prior to the examination according to the tenets of the Declaration of Helsinki.

**Table 1:** **Demographic and clinical information**.

	Patients with schizophrenia (*n* = 13)	Healthy controls (*n* = 24)	Relatives (*n* = 10)
Mean age (range)	33.8 (24–49)	37.9 (21–66)	62.3 (50–72)
Gender (male/female)	3/10	10/14	2/8
Mean years of education (range)	12.4 (9–18)	16.6 (10–23)	14.1 (12–20)
Mean duration of illness (SD)	11.9 (8.8)	n/a	n/a
Mean CED (SD)	435 (424)	n/a	n/a
Mean MRS (SD)	2.5 (4.0)	n/a	n/a
Mean PANSS positive (SD)	16.3 (5.4)	n/a	n/a
Mean PANSS negative (SD)	11.1 (4.5)	n/a	n/a
Mean PANSS total (SD)	53.8 (15.0)	n/a	n/a
Median visual acuity (IQR)	1.0 (0.5–1.0)	1.00 (0.7–1.0)	0.95 (0.7–1.0)

*SD, standard deviation; IQR, interquartile range; CED, chlorpromazine equivalent dose; MRS, modified Roger’s scale; PANSS, positive and negative syndrome scale; PANSS positive, positive symptoms subscale; PANSS negative, negative symptoms subscale; PANSS total, sum of all subscales*.

The groups significantly differed in age (see Table 1) because relatives (parents) were older compared to the other two groups (*F*_2,44_ = 16.6, *p* < 0.001), but patient ages were not significantly different from controls (Welch two-sample *t*-test: *t*_34,9_ = 1.03, *p* = 0.31). The groups had no significant gender differences (χ^2^ = 2.17, df = 2, *p* = 0.34). The groups were significantly different in amount of education; most prominently, the control group had four more years of education compared to patients (*F*_2,44_ = 10.4, *p* < 0.001). Visual acuity was not significantly different between groups (Wilcoxon rank sum test; patients vs. controls: *W* = 125, *p* = 0.48; patients vs. relatives: *W* = 56.5, *p* = 0.83; controls vs. relatives: *W* = 131, *p* = 0.66).

Eleven patients were being treated with atypical antipsychotics (usually risperidone or aripiprazole), one patient received both atypical and typical antipsychotics, and one patient was not taking any antipsychotic medication [chlorpromazine equivalent dosage (CED) in Table 1]. Five patients were taking additional medications: One patient was taking antidepressants (SSRI), two were taking both benzodiazepines and antidepressants (SSRI and tetracyclic), three were taking mood-stabilizers (sodium valproate), and one was taking an opioid. The positive and negative syndrome scale (PANSS) and the modified Rogers scale (MRS) were used to assess overall psychopathology (Table 1).

### 2.2 Apparatus

Eye movements of the dominant eye were recorded with a video-based infrared eye tracker (iView X HED-MHT, SMI, Germany) at a sampling rate of 200 Hz and a spatial resolution of 0.5°–1°. Stimuli were presented using our own software, which was based on PsychoPy (Peirce, 2008). Visual targets were presented in the center and periphery of the subject’s visual field to induce a large saccade. The visual targets were colored squares (red and yellow, 6 cm × 6 cm, 4.3° visual angle), Landolt rings (upward- or downward-oriented, 6 cm × 6 cm, 4.3° visual angle). All targets (central and peripheral) were presented at a viewing distance of 80 cm at individual eye height; peripheral targets at 55° in the left and right periphery. Further details and methodological aspects are described in our methods paper (Schwab et al., 2012).

### 2.3 Procedure

**Figure 1:**
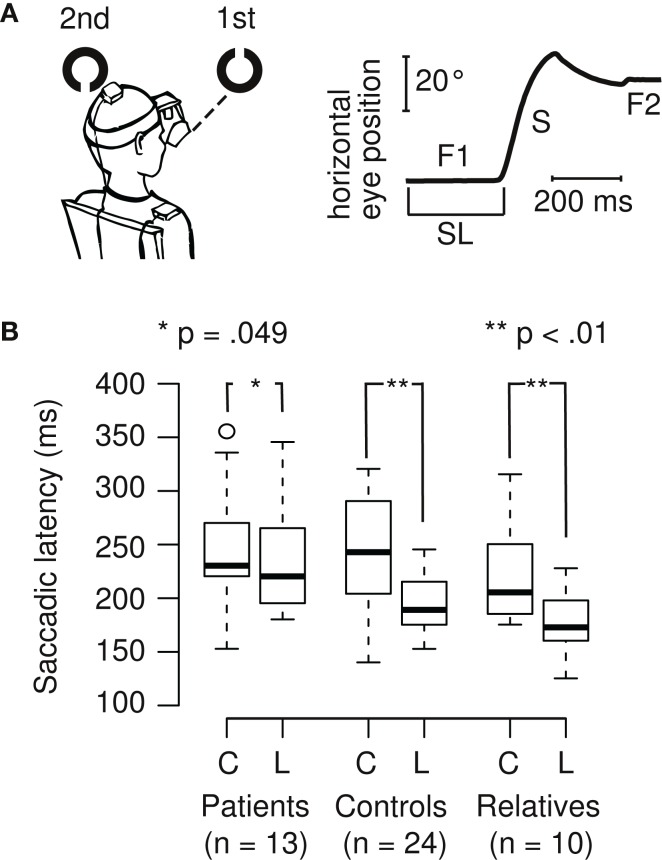
**(A)** Example of a Landolt trial and eye position data. When the first target disappears and the second target appears, the eye changes in horizontal position after a short delay, the saccadic latency (SL), which usually is around 200 ms. The initial fixation (F1) is followed by a saccade (S) and a subsequent fixation (F2) on the second target. **(B)** SL is shorter during the Landolt task (L, higher cognitive demand) compared to the color task (C, lower cognitive demand) in control subjects (26% decrease) and first-degree relatives (20% decrease). In patients with schizophrenia, this task difference is smaller (7% decrease), and also reached statistical significance.

First, visual acuity (Snellen chart), color vision (Ishihara test), visual dominance (Porta test), and handedness (Edinburgh inventory) were determined. All subjects were screened for eye diseases, diseases of the cervical vertebrae, neck and shoulder pain, drug abuse, and medication consumption. We used a peripheral recognition task (Schwab et al., 2012) to invoke large saccades. In the paradigm, a black fixation dot was presented. Then, the first target appeared in the same position, followed by a second target on either the left or right side (50% probability of each; Figure 1A). In the color task, we used color squares (red and yellow), and in the Landolt orientation task, we used Landolt rings (up- or downward orientation) as visual targets. The task was to determine whether these two objects (first and second target) were identical in terms of color (color squares) or orientation (Landolt rings). The subjects were instructed to move their eyes and head naturally as required and to make quick and accurate responses. In this context, we could precisely record large gaze shifts with our eye-head tracker consisting of both head and saccadic components, but in this paper we only focused on the saccadic latency time. The subjects pressed two buttons using their index (“Yes”) and middle (“No”) fingers of their dominant hands. In the experimental session, each subject performed 16 training trials, followed by 96 experimental trials spread across 3 blocks (32 trials per block). The trials involved color squares or Landolt rings (50% probability of each). For each subject, the conditions were randomly ordered and balanced within each block before the experiment started.

### 2.4 Analysis

Based on our previous study (Schwab et al., 2013), we performed a power analysis. Comparing the color with the Landolt task (within-subject factor), we found a significant saccadic latency reduction of 22.3% in the 12 healthy controls (color: 269 ± 36 ms; Landolt 209 ± 23 ms; paired *t*-test: *t*_11_ = 5.8, *p* = 0.0001). The effect size is large (Cohen’s *d* = 2), and a resulting power calculation based on this effect size, a significance level of 0.05, and a sample size of 10 subjects would result in a power of 0.99. Therefore, sample sizes of 10 and more subjects have sufficient statistical power to find significant effects (paired *t*-test power calculation; two-sided).

The data were preprocessed using our own custom MATLAB toolbox (Schwab et al., 2012). Eye recordings were transformed to visual angles (in degrees), low-pass filtered (750°/s), and smoothed (moving average over 20 ms). Saccades were detected using a velocity threshold algorithm with 60°/s, and 15°/s used as the onset and offset thresholds, respectively. SL values were then exported, and all statistics were performed in R (http://www.r-project.org/).

In order to classify the subjects based on the SL, we used Support Vector Machines (SVM, R package “e1071”). We trained to classify the subjects by using two values, the SL in the Landolt, and the SL in the color task. We compared the following classification accuracies: (1) classification of patients vs. controls, (2) classification of patients vs. relatives and controls, and (3) classification of patients and relatives vs. controls. In (1), we used *n* = 13 patients and a random sampling of *n* = 13 healthy controls (from a total of 24), in order to have equal group sizes. In (2), we used *n* = 13 patients and a random sampling of *n* = 13 of healthy controls and relatives (from a total of 34; 24 healthy controls and 10 relatives). In (3), we randomly sampled *n* = 13 patients and relatives (from a total 23, 13 patients and 10 relatives) and we randomly sampled *n* = 13 from the healthy controls (from a total of 24). Thus, our subsamples for the classification always contained a total 26 subjects (*n* = 13 per group) for all the three classification problems. We implemented leave-one-out-cross-validation, i.e., out training set always contained 25 subjects, and 1 subject was tested against the model prediction. This was implemented in a Monte Carlo run with 5 × 10^6^ iterations for each of the three classification problems, and mean specificity and sensitivity were calculated.

## 3 Results

Saccadic latencies (SL) in the different tasks (color and Landolt) were analyzed (Figures 1A,B). Patients had a small, significant reduction of only 6.5% between the two tasks (color: *M* = 248 ms, SD = 56 ms; Landolt: *M* = 232 ms, SD = 51 ms; two-sided paired *t*-test: *t*_12_ = 2.19, *p* = 0.049; *d* = 0.3). Controls had a large significant reduction of 26.2% in the Landolt task compared to the color task (color: *M* = 261 ms, SD = 100 ms; Landolt: *M* = 193 ms, SD = 27 ms; two-sided paired *t*-test: *t*_23_ = 3.26, *p* = 0.003; *d* = 0.9). First-degree relatives exhibited a large significant reduction of 19.5% in the Landolt task compared to the color task (color: *M* = 221 ms, SD = 49 ms; Landolt: *M* = 178 ms, SD = 30 ms; two-sided paired *t*-test: *t*_9_ = 3.58, *p* = 0.006; *d* = 1.1).

**Figure 2:**
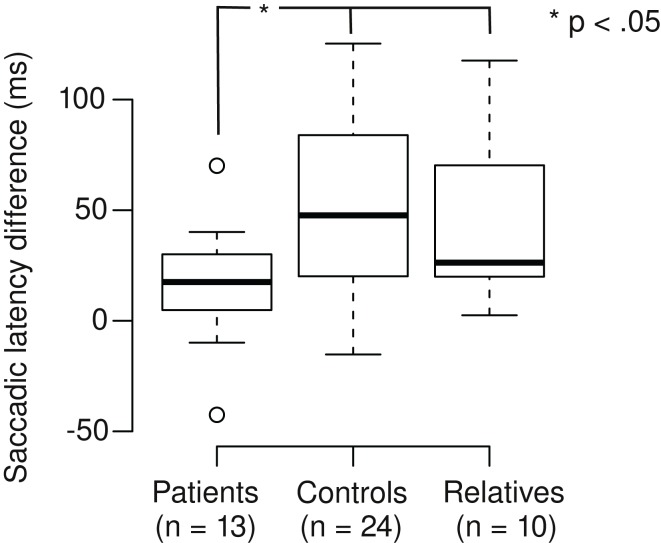
**Patients with schizophrenia exhibit smaller saccadic latency differences in the Landolt task (mean 16 ms) compared to control subjects (68 ms) and first-degree relatives (43 ms)**.

We calculated the saccadic latency differences (SLD) between the tasks by subtracting the SL in the Landolt task from the SL in the color task (Figure 2). Positive SLD values denote the extent of SL reduction in the Landolt task. SLD was 16 ms for patients, which was significantly shorter than controls (68 ms; Welch two-sample *t*-test, *t*_28,3_ = 2.35, *p* = 0.013; *d* = 0.6) and relatives (43 ms; Welch two-sample *t*-test, *t*_15,4_ = 1.90, *p* = 0.038; *d* = 0.8). There was no significant difference between controls and relatives (Welch two-sample *t*-test, *t*_31,8_ = 1.05, *p* = 0.15). In patients, there was no correlation of SLD with their CED (*r* = 0.17, *t*_11_ = 0.56, *p* = 0.59).

We compared changes in SL variance in the Landolt task compared to the color task (Figure 1B). Patients showed no significant change in the variance in the Landolt task (10% increase; Levene’s test, *F*_1,24_ = 0.03, *p* = 0.86). Controls had significantly smaller variance in the Landolt task (73% reduction; Levene’s test, *F*_1,46_ = 4.44, *p* = 0.041). Relatives showed no significant change in the variance during the Landolt task (38% reduction; Levene’s test, *F*_1,18_ = 1.46, *p* = 0.24).

**Figure 3:**
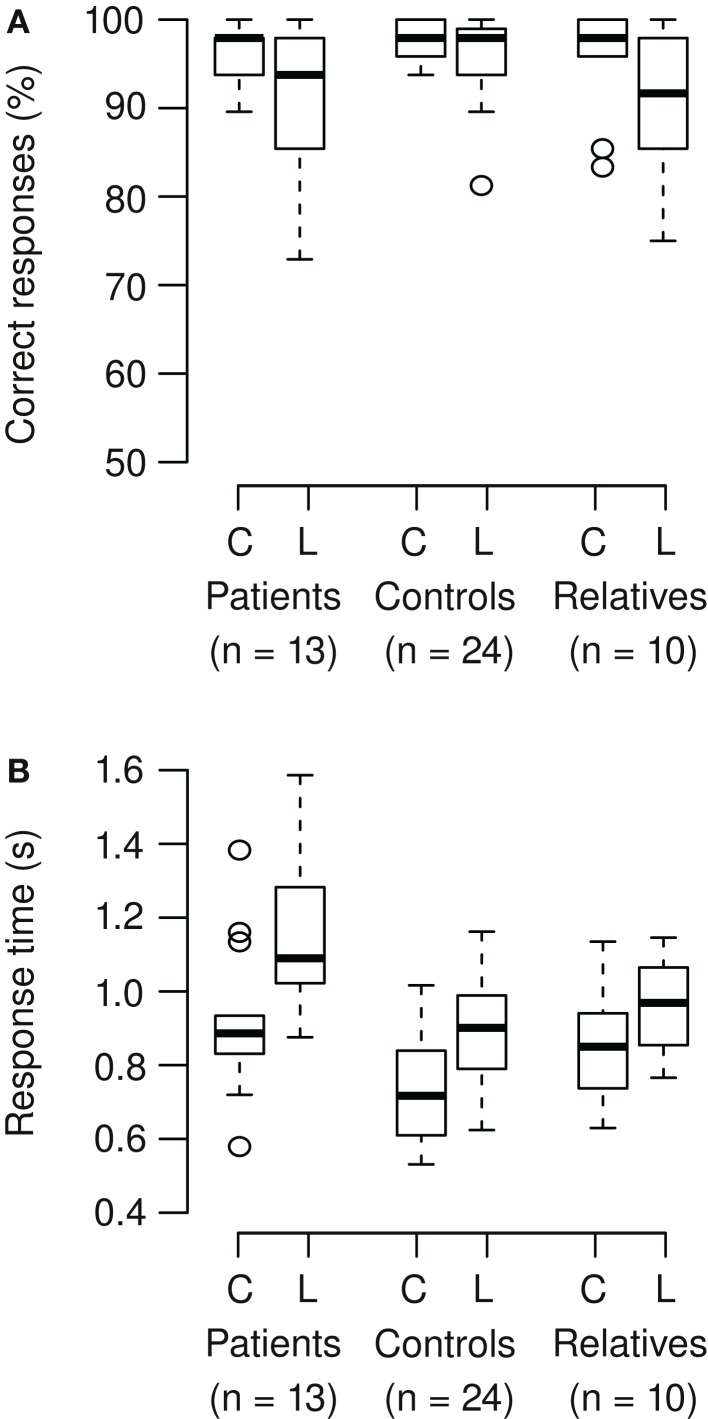
**(A)** Participants correctly responded in 95% of the trials (mean). In patients and relatives, accuracy of responses was lower in the Landolt (L) compared to the color condition (C), but there was only a trend toward significance (patients: *p* = 0.086; relatives: *p* = 0.050). **(B)** The Landolt task produced longer response times across groups (*p* < 0.001). The patients exhibited longer response times across tasks (*p* < 0.001). Also, the relative increase of reaction time in the Landolt task was larger in patients compared to the other two groups (significant interaction of task × group; *p* = 0.002).

Correct responses and response times were analyzed (Figures 3A,B). On average, participants correctly responded in 95% of the trials. In patients and relatives, accuracy of responses was lower in the Landolt compared to the color condition, but this was not significant (Wilcoxon signed rank test; patients: *V* = 70.5, *p* = 0.086; relatives: *V* = 39.5, *p* = 0.050). The Landolt task produced longer response times across groups compared to the color task (mean increase 22%). The patients exhibited longer response times across tasks compared to the other groups (mean increase 24%). Also, the relative increase of reaction time in the Landolt task was larger in patients (28%) compared to the other two groups (12%). These results were confirmed by ANOVA (main effect group: *F*_2,44_ = 8.65, *p* < 0.001; main effect task: *F*_1,44_ = 155, *p* < 0.001; interaction task × group: *F*_2,44_ = 7.01, *p* = 0.002).

**Figure 4:**
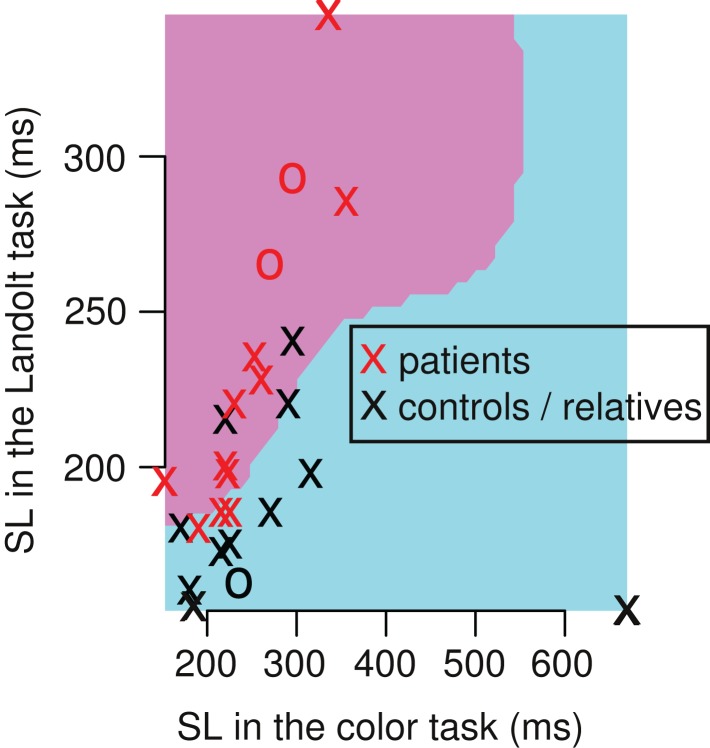
**Single example of a Monte Carlo iteration of the support vector machines (SVM) that classify between patients vs. relatives/healthy controls**. The classification had a mean sensitivity of 61%, and a mean specificity of 62% over 5 × 10^6^ iterations. In each iteration, we used 26 subjects (13 patients, 13 relatives/healthy controls) and implemented a leave-one-out-cross-validation (training-set: *n* = 25, test-set: *n* = 1). The single iteration above shows a radial kernel classifier (pink and blue area) of the training set. This model classifies subjects within the pink area as patients, and subjects within the blue area as controls/relatives. Note that the pink area covers longer SL in the Landolt task compared to the color task, and the blue area is associated with shorter SL in the Landolt task compared to the color task.

In the context that SL patterns may be useful for classification of the subjects, we used SVM with leave-one-out-cross-validation in a Monte Carlo experiment. We found that the SVM could classify the subjects (patients vs. relatives and healthy controls) with a mean sensitivity of 61%, and a specificity of 62% (Figure 4). The confusion matrix includes eight true positives, five false positives, eight true negatives, and five false negatives. Taken together, 16 subjects (62%) were correctly classified in average, 10 were incorrectly classified (38%). Performing other classifications resulted into lower accuracies: patients vs. healthy controls showed a mean sensitivity of 57%, and a specificity of 53%, and patients and relatives vs. healthy controls showed a mean sensitivity of 42%, and a specificity of 40%.

We tested five patients, two who were taking benzodiazepines, and three who were taking valproic acid for a potential medication effect of increased SL. During the color task, the five patients taking the medication mentioned above had a mean SL of 239 ms (SD = 56 ms), and the eight patients not taking this medication had a mean SL of 255 ms (SD = 59 ms), which was not a significant difference (Welch two-sample *t*-test: *t*_9_ = 0.49, *p* = 0.64). In the Landolt task, the five patients taking the medication had a mean SL of 226 ms (SD = 68 ms), and the eight patients not taking this medication had a mean SL of 236 ms (SD = 42 ms), which was not a significant difference (Welch two-sample *t*-test: *t*_5,9_ = 0.27, *p* = 0.79).

In summary, the Landolt task caused (1) shorter SL and smaller within-group variability in controls and first-degree relatives compared to the patients, (2) longer response times consistently across groups, and (3) a relatively larger increase in response times of patients compared to the other two groups (interaction group × task). Further, classification based on SL works better merging the relatives and the controls and then classify into patients vs. healthy controls/relatives compared to merging the patients and relatives and then classy between patients and relatives vs. healthy controls.

## 4 Discussion

It has been shown that SL are strongly task dependent: higher cognitive demand causes shorter SL, while lower cognitive demand causes longer SL (Trottier and Pratt, 2005; Guyader et al., 2010). The present study demonstrates that SL modulation indeed fails in patients with schizophrenia but not in first-degree relatives during the two tasks of different cognitive demand. First-degree relatives and controls showed shorter SL in the Landolt task, and longer SL in the color task, consistent with previous findings (Trottier and Pratt, 2005; Guyader et al., 2010). The lack of SL modulation in patients may be caused by impaired top-down saccade generation in schizophrenia. Multiple brain areas are involved in the accurate execution of saccadic eye movements, most importantly the frontal eye field (FEF), which controls visual attention (Schall, 2004; Roux et al., 2014).

The fact that impaired SL modulation was not found in first-degree relatives does not support SL as possible trait marker. We found task-dependent modulation of SL in relatives and healthy controls, but not to the same extent in the patients. Also, our SVM classifier exhibited best accuracies during classification into a patient vs. a merged relatives/healthy control group. Thus, our data indicate impaired SL modulation may be a state marker, but a potential relation to psychopathology has yet to be studied. To take full advantage of this, however, further investigations, including longitudinal studies, will be required in order to relate objective measures such as SL to treatment outcomes or psychopathology scales (Walther et al., 2009). The results of this study are consistence with the literature showing that not all oculomotor parameters fully meet the criteria of an endophenotype, for example, antisaccade impairments (Levy et al., 2008). An explanation is that the various impaired oculomotor parameters in schizophrenia and the different experimental tasks (antisaccade, visual exploration, smooth pursuit, etc.) relate to different neurocognitive deficits arising from different brain circuits. Scanpath patterns likely relate to visual attention, fixations to visual memory, and antissaccades and SL to the control of executive and inhibitory frontal functions. Different parameters highlight a specific aspect of visual behavior and oculomotor control and contribute to the understanding of the underlying pathophysiology of schizophrenia.

Variance of SL decreased in the Landolt task compared to the color task, but this was only significant for the control group, indicating that this group became more homogeneous in their performance when faced with increased cognitive demand.

Performance data confirmed that the two tasks were of different cognitive demand. Landolt tasks produced fewer correct responses and longer response times. A possible explanation is that the Landolt orientation task required more foveal vision and mental rotation, while the color condition required peripheral vision during color detection. This is supported by larger saccade amplitudes found in the Landolt task than in the color task in our previous study (Schwab et al., 2013).

We now address some limitations of the study. First, most patients were taking psychoactive medication, which can affect oculomotor functions. For example, it was shown that atypical antipsychotic medication can impair performance in smooth pursuit (Lencer et al., 2008). On the other hand, it was found that antipsychotic medication can improve eye movement control (Burke and Reveley, 2002). Therefore, the overall effect of atypical antipsychotics on eye movement performance is ambiguous. Atypical antipsychotics have been suggested to even improve oculomotor markers, but evidence is lacking (Schmechtig et al., 2013). Three patients were taking valproic acid, and two were taking benzodiazepines, which was both associated with an increase of saccadic latency (Reilly et al., 2008; Larrison et al., 2011). However, the five patients who were taking valproic acid or benzodiazepines had no increase in SL compared to the other patients. Also, we observed that SL in patients were not generally increased compared to controls, which argues against a general slowing effect due to medication. Also, we could not find a correlation between SL and CED. Thus, even though our data suggest that the effects are not confounded by the type or the amount of medication, impaired saccadic latencies modulation observed in patients cannot be attributed to illness effects alone, thus the medication influence has to be further studied.

Second, it is unclear whether our findings are specific to schizophrenia, since we have not studied another pathological group (e.g., patients with mood disorders). Even though a recent review concluded that findings concerning anxiety and mood disorders have failed to support oculomotor impairments in these diseases (Toh et al., 2011), abnormal eye movements, but not specifically abnormal saccadic latencies, have been found in bipolar disorder (Bestelmeyer et al., 2006). In the context that symptoms and the brain systems affected in different psychiatric conditions can overlap, it would be surprising to find abnormal eye movements only in schizophrenia. Nevertheless, eye movements seem to be a promising instrument for diagnosis and classification of patients with schizophrenia (Benson et al., 2012), and eye movement should in future studies be tested to classify different subtypes in schizophrenia, similar as eye movement can distinguish subtypes in Parkinson’s disease and dementia (Willard and Lueck, 2014). The fact that the first-degree relatives are significantly older than the other two groups is not a real limitation of the study as we found significant saccadic latency modulation in the first-degree relatives, likewise as the younger healthy controls. The elderly group seems to perform as well as the younger healthy controls in view of saccadic latency modulation. Therefore, we conclude aging does not seem to affect saccadic latency adaption toward different tasks.

SL modulation during tasks with lower and higher cognitive demand is an important attentional feature to adapt to a changing environment by allowing the brain to save resources during low demand and to optimize performance during higher demand. The SL deficiencies found in patients with schizophrenia, but not in first-degree relatives, may be a state marker associated to their clinical condition. SL impairments may have their greatest impact during times of concentration. In this way, oculomotor deficits, may influence higher-order cognition (Butler and Javitt, 2005). For example, they could lead to difficulties determining which stimuli are relevant in the situations of daily life that require higher attentional resources.

## Author Contributions

SS and AA designed the experiments, MJ performed the experiments, and SS analyzed the data. SS wrote the first draft of the paper. All authors contributed to the final version and have approved the final manuscript.

## Conflict of Interest Statement

The authors declare that the research was conducted in the absence of any commercial or financial relationships that could be construed as a potential conflict of interest.
